# Dual effects of the alternative spliced RIG-I isoform PTIR1 on host antiviral defense and immune homeostasis

**DOI:** 10.1038/s41419-025-08159-x

**Published:** 2025-11-10

**Authors:** Jia Song, Wenyu Tian, Lulu Liu, Xuyang Zhao, Wei Zhao, Dan Lu

**Affiliations:** 1 Department of Dental Materials & Dental Medical Devices Testing Center, Peking University School and Hospital of Stomatology, Beijing, PR China; 2 Institute of Systems Biomedicine, School of Basic Medical Sciences, NHC Key Laboratory of Medical Immunology, Beijing Key Laboratory of Tumor Systems Biology, Peking University, Beijing, PR China; 3 Department of Geriatric Dentistry, Peking University School and Hospital of Stomatology, Beijing, PR China; 4 Department of Clinical Laboratory, China-Japan Friendship Hospital, Beijing, PR China

**Keywords:** Autoimmunity, RIG-I-like receptors, Inflammation

## Abstract

Efficient pathogen recognition must be tightly regulated to prevent immunopathology. Here, we identify PTIR1, a primate-specific splice variant of *DDX*58 lacking exon 4 and encoding a truncated RIG-I isoform, as a negative regulator of innate immune signaling. PTIR1 is selectively induced upon viral infection or interferon (IFN) stimulation, and its ectopic expression via adenoviral delivery broadly suppresses inflammatory responses in vivo. Mechanistically, PTIR1 activates the deubiquitinase UCHL5 to limit STAT1 ubiquitination at lysine 525, thereby impairing STAT1 nuclear translocation and dampening type I and type II IFN responses. Accordingly, in a model of autoimmune hepatitis, PTIR1 restricts IFN-γ-driven inflammation, and in a viral infection model, it attenuates type I IFN responses. PTIR1 also modulates RIG-I signaling by interfering with its dimerization, reducing its ubiquitination, and disrupting its interaction with MAVS, thereby limiting RIG-I-mediated antiviral recognition and facilitating viral immune evasion. These findings identify PTIR1 as an inducible post-transcriptional checkpoint that fine-tunes antiviral and inflammatory signaling to preserve tissue integrity.

## Introduction

Host innate immunity plays a critical role in defending against viral infection through pattern recognition receptors (PRRs), such as retinoic acid–inducible gene I (RIG-I) and melanoma differentiation–associated protein 5 (MDA5), which detect viral nucleic acids and initiate downstream signaling [1–3]. Upon RNA sensing, RIG-I translocates to the mitochondria and activates MAVS, leading to TBK1 recruitment, IRF3 phosphorylation, and type I interferon (IFN-I) induction. Secreted IFN-I engages the JAK-STAT pathway to stimulate expression of interferon-stimulated genes (ISGs), which restrict viral replication and establish an antiviral state [4, 5]. While essential for antiviral defense, IFN-I signaling must be tightly regulated, as persistent or excessive activation can drive chronic inflammation and autoimmune disorders such as systemic lupus erythematosus, rheumatoid arthritis, and multiple sclerosis [6–8].

Signal transducer and activator of transcription 1 (STAT1) is a key transcription factor downstream of both type I and type II IFNs. Its activity is primarily regulated by phosphorylation at tyrosine 701 and serine 727, but other post-translational modifications, such as methylation and ubiquitination, also influence its nuclear localization and transcriptional function [9–12]. In particular, the role of ubiquitination in modulating STAT1 activity remains incompletely understood.

Alternative splicing provides a versatile mechanism for diversifying immune responses. Viral infections reshape host splicing programs, giving rise to novel isoforms that influence cell fate and immune signaling [13, 14]. Conversely, viruses hijack host splicing machinery to evade detection [15]. Despite its importance, the functional impact of alternatively spliced isoforms in innate immune regulation remains largely undefined.

Promoter of Tumor Immune Resistance 1 (PTIR1) is a primate-specific splice variant of *DDX58*, which encodes RIG-I. Lacking exon 4, PTIR1 produces a truncated 142-amino-acid protein and has been previously implicated in impairing antigen presentation and promoting immune escape in colorectal cancer [16]. However, its physiological role in host antiviral and inflammatory responses is unknown.

Here, we identify PTIR1 as an inducible negative regulator of innate immunity. PTIR1 expression is triggered by viral infection or IFN stimulation, and its overexpression limits tissue damage in murine models of autoimmune hepatitis and acute kidney injury. Mechanistically, PTIR1 promotes STAT1 interaction with the deubiquitinase UCHL5, suppressing STAT1 ubiquitination at lysine 525 and blocking its nuclear translocation independent of phosphorylation. In parallel, PTIR1 associates with RIG-I, inhibits its ubiquitination at lysine 172, and disrupts MAVS binding, thereby attenuating antiviral signaling. While protective in inflammatory contexts, PTIR1 compromises antiviral defense by dampening IFN responses. These findings establish PTIR1 as a post-transcriptional immune checkpoint that fine-tunes STAT1 and RIG-I activity to maintain immune homeostasis.

## Results

### PTIR1 restricts inflammatory damage in vivo

Our previous work identified PTIR1 as a primate-specific splice variant of *DDX58* that lacks exon 4, resulting in a truncated 142-amino-acid protein associated with poor prognosis in colorectal cancer (Supplemental Fig. 1A) [16]. To assess its subcellular localization, we cloned full-length RIG-I, PTIR1, and an active N-terminal truncation of RIG-I (RIG-I_1-284_) into the pEGFP-N1 vector. As expected, RIG-I_1-284_ localized to mitochondria due to the absence of its C-terminal autorepression domain, whereas both PTIR1 and full-length RIG-I were predominantly cytosolic (Supplementary Fig. 1B).

**Fig. 1:**
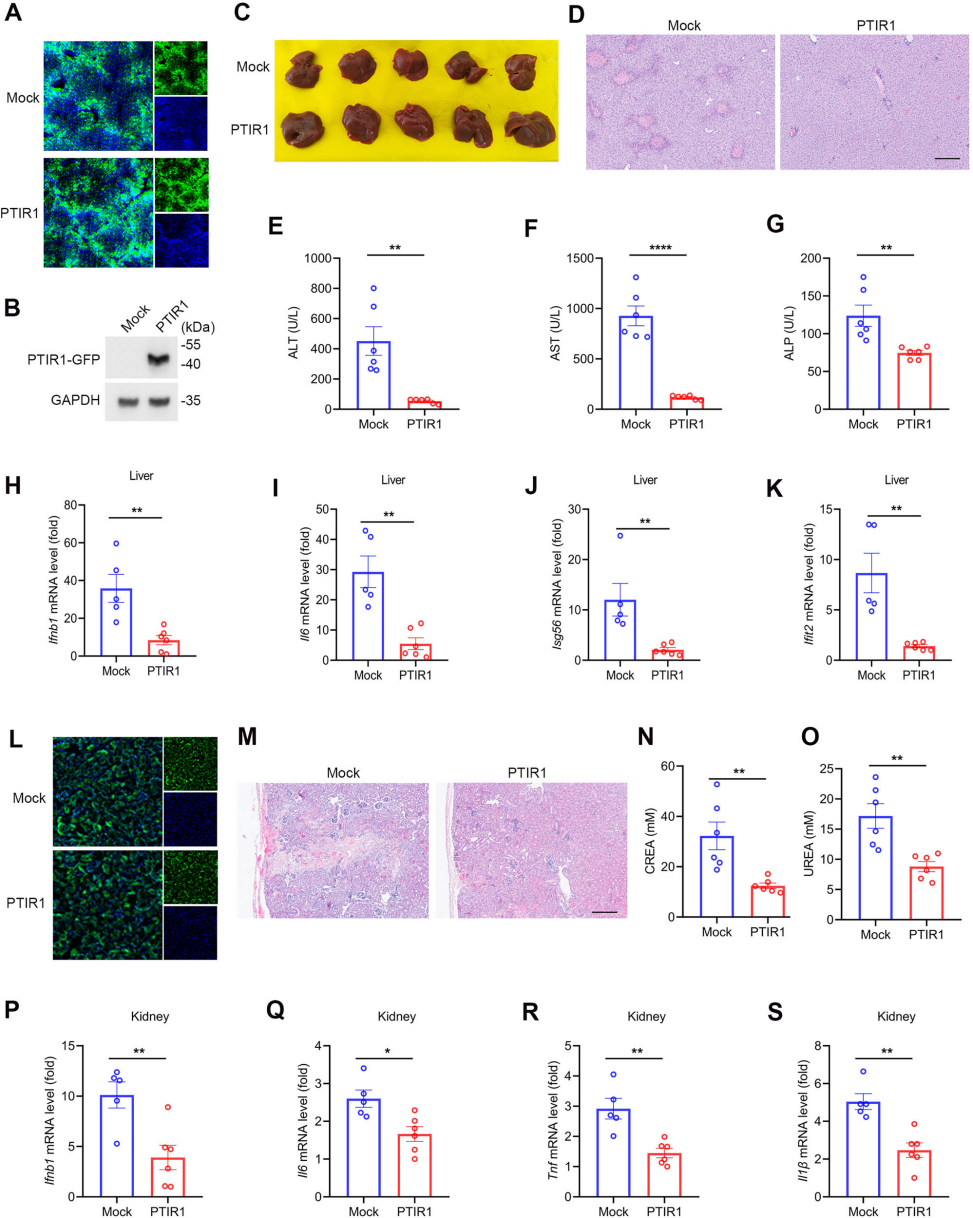
PTIR1 attenuates host acute inflammatory damage. **A** Immunofluorescence analysis of PTIR1 expression in mouse liver. **B** Immunoblot analysis of PTIR1 expression in mouse liver. **C**, **D** Gross evaluation and histology analysis of mice livers 24 h after Con A administration. **E–G** Mice serum was collected and blood biochemistry test was performed. ALT, Alanine aminotransferase; AST, Aspartate aminotransferase; ALP, alkaline phosphatase (n = 6, mean ± s.e.m., **P < 0.01, ****P < 0.0001, unpaired Student’s *t*-test). **H–K** Quantitative real-time PCR analysis of the transcription of indicated genes in mice livers with or without PTIR1 24 h after Con A administration (Mock, n = 5 mice; PTIR1, n = 6 mice, mean ± s.e.m., **P < 0.01, unpaired Student’s *t*-test). The primers used for quantitative real-time PCR have been deposited in Supplemental Table 1. **L** Immunofluorescence analysis of PTIR1 expression in the mouse kidney. **M** Histology analysis of mice kidneys 24 h after AKI. **N**, **O** Mice serum was collected and blood biochemistry test was performed (n = 6, mean ± s.e.m., **P < 0.01, unpaired Student’s *t*-test). **P–S** Quantitative real-time PCR analysis of the transcription of indicated genes in mice kidneys with or without PTIR1 24 h after AKI (Mock, n = 5 mice; PTIR1, n = 6 mice, mean ± s.e.m., *P < 0.05, **P < 0.01, unpaired Student’s *t*-test). The primers used for quantitative real-time PCR have been deposited in Supplemental Table 1.

To examine its biological function in vivo, we used adenoviral vectors to deliver PTIR1 or a mock control into mice. As shown in Supplementary Fig. 1C, fluorescence imaging demonstrated that PTIR1 expression was primarily restricted to the liver and kidney. Overexpression of PTIR1 did not affect basal physiological parameters. Since PTIR1 is inducible by both type-I and type II interferons (IFNs), we assessed its function under inflammatory conditions. We employed two models of acute inflammation, the concanavalin A induced autoimmune hepatitis model and the ischemia-reperfusion-induced acute kidney injury model. In the liver inflammation model, PTIR1 expression was validated by tissue imaging and immunoblotting (Fig. 1A, B). Mice expressing PTIR1 showed reduced liver damage, with improvements in gross morphology and histological architecture (Fig. 1C, D). Serum concentrations of alanine aminotransferase (ALT), aspartate aminotransferase (AST) and alkaline phosphatase (ALP) were significantly lower in the PTIR1 group compared to controls (Fig. 1E–G). Hepatic levels of inflammatory cytokines were also decreased in the PTIR1 group (Fig. 1H–K and Supplementary Fig. 1D, E).

Adenovirus-mediated PTIR1 expression was also detected in the kidney (Fig. 1L). In the kidney inflammation model, PTIR1 overexpression led to reduced vascular congestion and tubular injury (Fig. 1M) and preserved renal function (Fig. 1N, O). Expression of pro-inflammatory cytokine transcripts in the kidney was also reduced in the PTIR1 group (Fig. 1P-S and Supplemental Fig. 1F, G). Together, these results indicate that PTIR1 functions as a suppressor of acute inflammatory responses in vivo.

### PTIR1 curbs immune response by limiting STAT1 activity

**Fig. 2:**
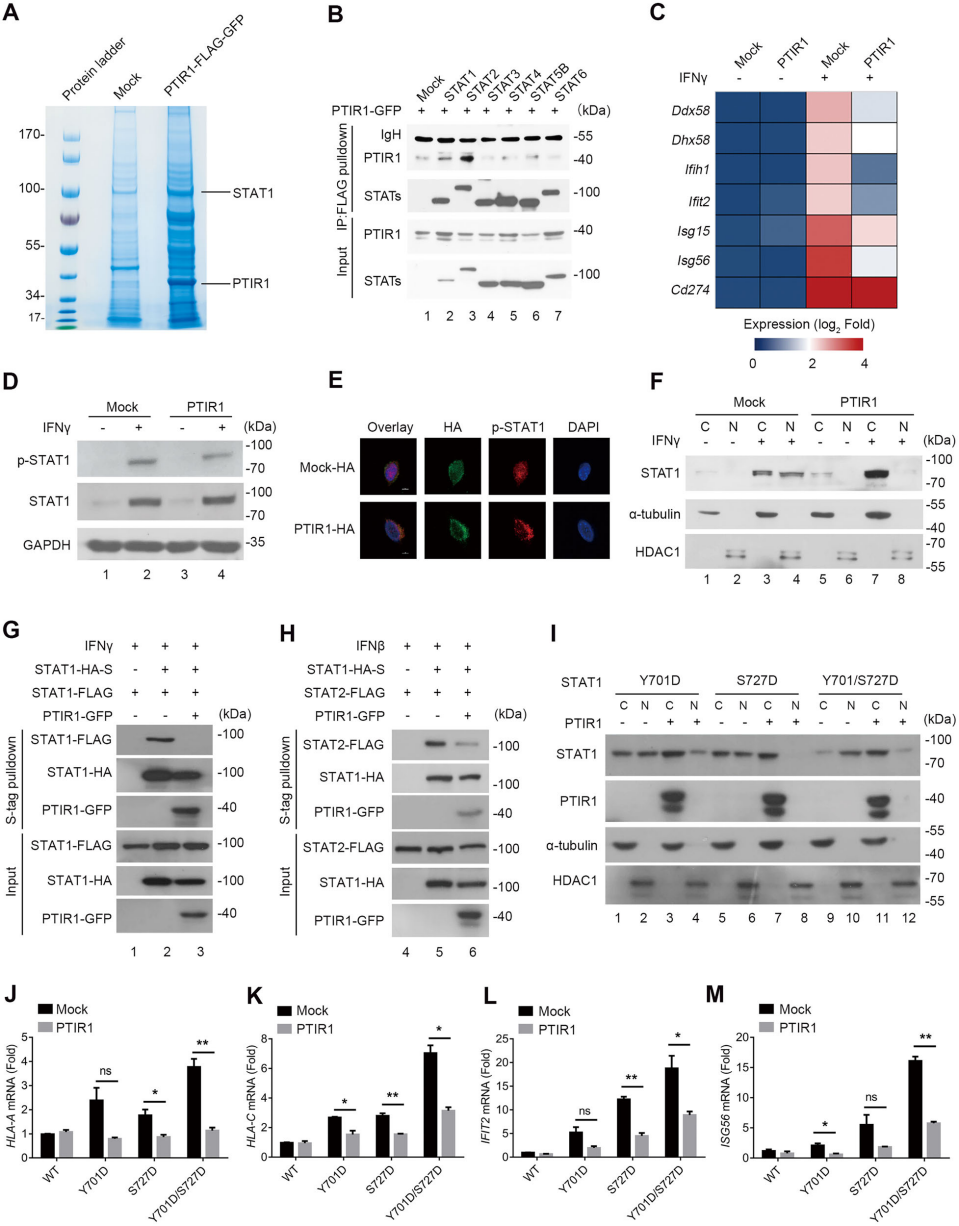
PTIR1 suppresses STAT1 activation and blocks its nuclear translocation. **A** MS analysis of PTIR1-associated proteins after FLAG pulldown assay in HEK293T cells under SeV infection. Associated proteins were shown. **B** Interaction with PTIR1 and STAT family members. Indicated plasmids were transfected into HEK293T cells, followed by co-immunoprecipitation with anti-FLAG antibody. **C** Transcription of interferon stimulated genes was analyzed by quantitative real-time PCR in Mock and PTIR1 expressing HEK293T cells stimulated by IFNβ or not. The primers used for quantitative real-time PCR have been deposited in Supplemental Table 1. **D** Effect of PTIR1 on phosphorylation of STAT1 in LLC cells upon IFNγ (100 ng/mL) treatment, tested by western blot with anti-phospho-STAT1 antibody. **E** Images of immunofluorescence staining for HA (PTIR1) and p-STAT1 in LLC cells with IFNγ (100 ng/mL) treatment by confocal microscope. The scale bars represent 10 μm. **F** Subcellular localization of STAT1 in LLC cells expressing Mock and PTIR1 treated by IFNγ (100 ng/mL) or not. C, cytosol, N, nuclear. **G**, **H** Effect of PTIR1 on STAT1 homodimerization and heterodimerization of STAT1 and STAT2 during interferon stimulation. HEK293T cells were transfected with indicated plasmids and treated by IFNγ (100 ng/mL) or IFNβ (100 ng/mL), respectively, followed by co-immunoprecipitation with S-tag. **I** Subcellular localization of variants STAT1 co-transfected with PTIR1 or not in HEK293T cells. C, cytosol, N nuclear. **J**–**M** Quantitative real-time PCR analysis of the transcription of indicated genes in HEK293T cells expressing wildtype (WT) or mutant STAT1 with or without PTIR1 (n = 2 biological replicates, mean ± s.e.m., ns, not significant (*P* > 0.05), *P < 0.05 and **P < 0.01, unpaired Student’s *t*-test). The primers used for quantitative real-time PCR have been deposited in Supplemental Table 1.

To uncover the molecular mechanism by which PTIR1 suppressed the immune response, we first used immunoprecipitation coupled with mass spectrometry and identified the transcription factor STAT1 as a PTIR1-associated protein (Fig. 2A). Co-immunoprecipitation (Co-IP) assay further confirmed that PTIR1 selectively interacts with STAT1 and STAT2, but not with other members of the STAT family (Fig. 2B). Given the crucial role of STAT1 in IFN signaling, we next investigated the impact of PTIR1 on STAT1 transcriptional activity. Indeed, in PTIR1-expressing cells treated with either type-I or type-II IFN, the transcription of STAT1 target genes, such as *IFIH1, IFIT2, ISG15, and ISG56*, was significantly downregulated (Fig. 2C). Since STAT1 phosphorylation and nuclear import are essential for the propagation of IFN signaling, we assessed the effects of PTIR1 on STAT1 activation following IFNγ stimulation. Although the phosphorylation levels of STAT1 remained identical in the presence of PTIR1, we observed that PTIR1 effectively blocked STAT1 nuclear translocation, as measured by confocal microscopy (Fig. 2D, E). This was further corroborated by subcellular fractionation assays (Fig. 2F). Additionally, overexpression of PTIR1 impaired the homo- or heterodimerization of STAT1 with STAT2 in response to type-II or type-I interferon stimulation, respectively (Fig. 2G, H). Notably, the inhibitory effects of PTIR1 on STAT1 nuclear translocation and transcriptional activity were observed even with STAT1 phosphorylation-mimic mutants, indicating that PTIR1 exerts its effects in a phosphorylation-independent manner (Fig. 2I-M). Together, these findings demonstrate that PTIR1 efficiently blocks STAT1 nuclear translocation and inhibits IFN signaling.

### PTIR1 activates the deubiquitinase UCHL5 and inhibits STAT1 ubiquitination

**Fig. 3:**
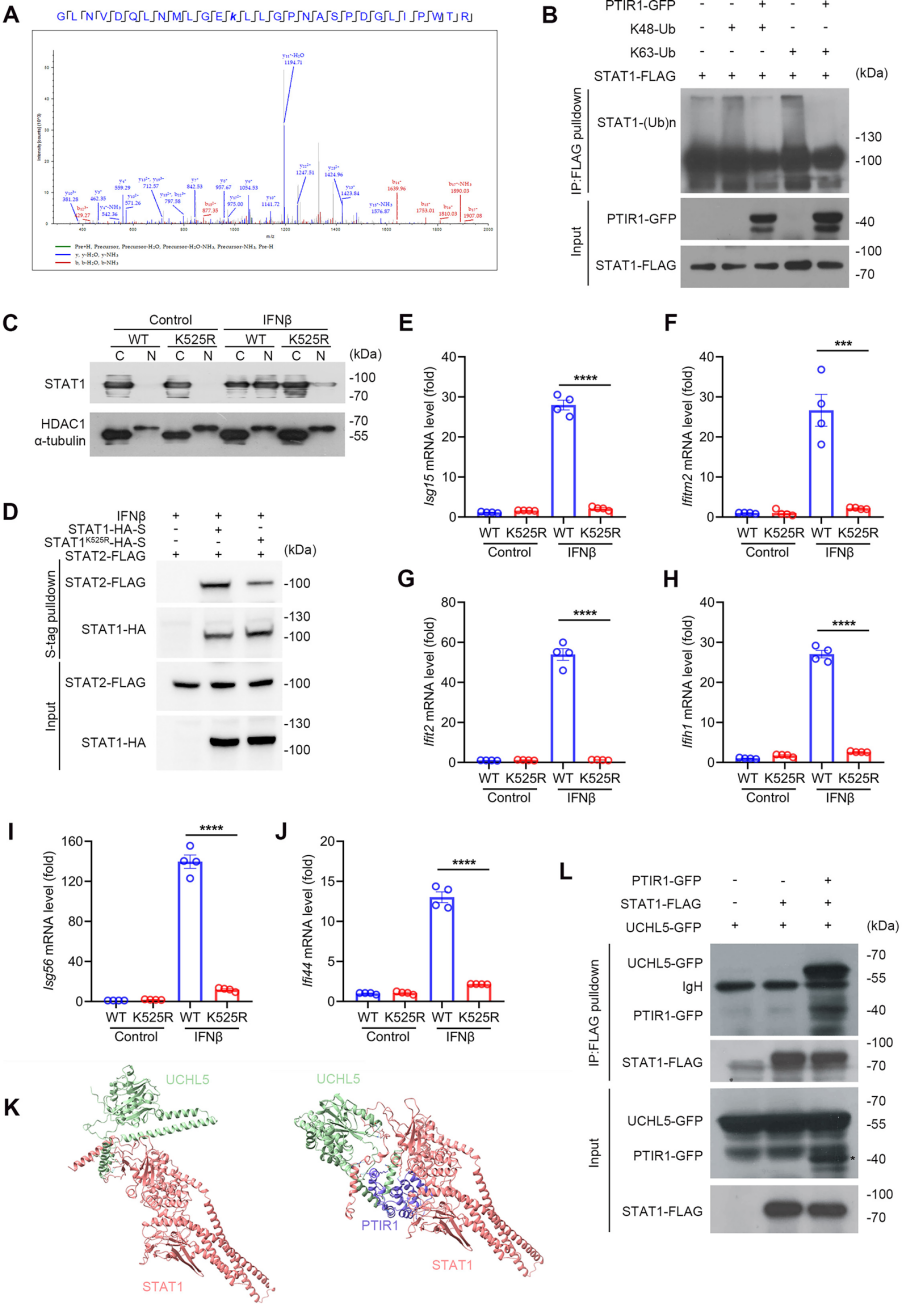
PTIR1 inhibits STAT1 ubiquitination by recruiting and activating UCHL5. **A** MS analysis of ubiquitination of STAT1 Lysine 525. STAT1-FLAG was co-transfected with or without ectopically expressing PTIR1-GFP into HEK293T cells, then purified with anti-FLAG M2 affinity gel and mass spectrum was performed to analyze STAT1 modifications. **B** Immunoprecipitation analysis of ubiquitination of STAT1 in HEK293T cells co-transfected with indicated plasmids. **C** The role of K525R mutant in STAT1 subcellular location. HEK293T cells were transfected with STAT1^WT^ or STAT1^K525R^ expression plasmids. After IFNβ (100 ng/mL) stimulation for 24 h, cells were subjected to nucleus-cytoplasm extraction. Immunoblot analysis was performed by anti-STAT1 antibody. **D** Effect of K525R mutation on STAT1 dimerization during interferon stimulation. HEK293T cells were transfected with indicated plasmids and treated by IFNβ (100 ng/mL), followed by co-immunoprecipitation with S-tag. **E–J** Quantitative real-time PCR analysis of the transcription of indicated genes in BMDM cells expressing STAT1^WT^ or STAT1^K525R^ in presence or absence of IFNβ (100 ng/mL) stimulation for 24 h (n = 4 biological replicates, mean ± s.e.m., ***P < 0.001 and ****P < 0.0001, unpaired Student’s *t*-test). The primers used for quantitative real-time PCR have been deposited in Supplemental Table 1. **K** Structural analysis of UCHL5 and STAT1/PTIR1 complex predicted by AlphaFold3. **L** The interaction between STAT1 and UCHL5 in presence of PTIR1 or not. HEK293T cells were transfected with indicated plasmids and were subjected to immunoprecipitation with anti-FLAG antibody, followed by western blot analysis.

Given the importance of post-translational modification (PTM) in regulating STAT1 nuclear accumulation, we employed immunoprecipitation coupled with mass spectrometry to examine the post-translational status of STAT1 in the presence of PTIR1. Consistent with our immunoblotting data (Fig. 2D), PTIR1 had minimal effects on STAT1 phosphorylation. Notably, STAT1 ubiquitination at lysine 525 was induced upon viral infection, but this modification was suppressed by PTIR1 (Fig. 3A). Co-IP assay further confirmed that PTIR1 inhibits STAT1 ubiquitination (Fig. 3B). To determine the functional consequences of this modification, we generated a STAT1^K525R^ mutant, in which lysine 525 was replaced with arginine. Relative to wild-type STAT1, the K525R mutant displayed substantially impaired nuclear translocation in response to IFNβ stimulation (Fig. 3C). S-tag pulldown assays revealed that the K525R mutation also reduced STAT1-STAT2 interaction under IFNβ stimulation (Fig. 3D). Furthermore, transcriptional activation of ISGs, including *Ifitm2* and *Ifih1*, was significantly diminished in STAT1^K525R^-expressing cells compared with wild-type controls following IFNβ stimulation (Fig. 3E–J and Supplementary Fig. 2A–C). These findings are consistent with the suppressive effects of PTIR1 on STAT1 activity.

Although PTIR1 is an alternatively spliced isoform of RIG-I lacking canonical deubiquitinase motifs, our previous work showed that PTIR1 interacts with UCHL5, unlike full-length RIG-I [16]. We hypothesized that PTIR1 facilitates STAT1 deubiquitination by recruiting UCHL5. Structure prediction using AlphaFold3 indicated that PTIR1 binds the DNA-binding domain of STAT1 (Supplementary Fig. 3A). When UCHL5 was incorporated into the model, its association with STAT1 appeared weak in the absence of PTIR1, but was markedly enhanced when PTIR1 was present (Fig. 3K). This effect was validated by the Co-IP assay, which showed that PTIR1 significantly promoted the interaction between STAT1 and UCHL5 (Fig. 3L). These results suggest that PTIR1 acts as a scaffold to enhance UCHL5-mediated deubiquitination of STAT1, thereby inhibiting STAT1 nuclear translocation and downstream signaling.

### PTIR1 ameliorates IFN-γ-driven liver inflammation in autoimmune hepatitis

**Fig. 4:**
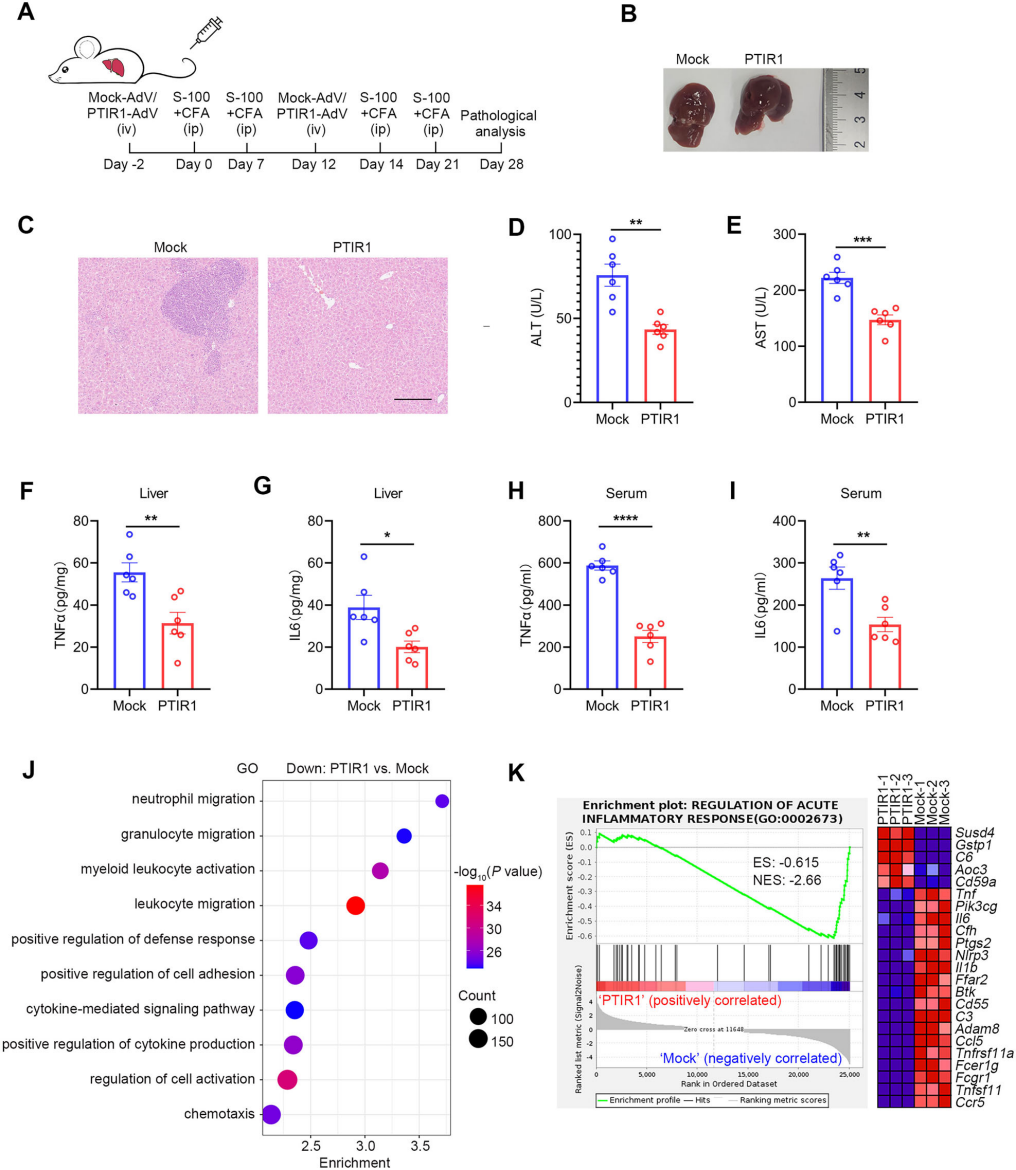
PTIR1 alleviates S100-induced autoimmune hepatitis in mice. **A** Graphic illustration of the study design. Mock or PTIR1 was first overexpressed in mouse liver through adenovirus infection, and S-100-induced AIH model was established. **B**, **C** Gross evaluation and histology analysis of mice livers. The scale bars represent 200 μm. **D**, **E** Mice serum was collected and blood biochemistry test was performed. ALT, Alanine aminotransferase; AST, Aspartate aminotransferase (n = 6, mean ± s.e.m., **P = 0.0011, ***P = 0.0002, unpaired Student’s *t*-test). (**F**–**I**) ELISA detection of inflammatory cytokines in mice livers or blood (n = 6, mean ± s.e.m., *P = 0.0146, **P = 0.0056 (TNFα in liver), **P = 0.0060 (IL6 in blood), ****P < 0.0001, unpaired Student’s *t*-test) (**J**) Genes that were significantly downregulated in PTIR1 expressing liver, compared with Mock liver, were analyzed using DAVID with GO terms. **K** GSEA of genes expressed in mouse liver in AIH model, in the presence or absence of PTIR1 (n = 3 biological replicates). ES enrichment score, NES normalized enrichment score.

Having observed that PTIR1 inhibits STAT1-mediated type I interferon (IFN) signaling and attenuates type II IFN (IFN-γ) responses in vitro, we next investigated whether PTIR1 overexpression could mitigate IFN-γ-driven liver inflammation in vivo. To test this, we used an S100-induced autoimmune hepatitis (AIH) mouse model (Fig. 4A). S100-immunized control mice developed severe hepatic pathology, including hepatocyte swelling and rupture, accompanied by dense inflammatory infiltrates in portal regions. In contrast, PTIR1-overexpressing mice showed markedly reduced immune-cell infiltration and only mild hepatocellular enlargement (Fig. 4B, C). Consistent with these histological improvements, PTIR1-overexpressing mice had significantly lower serum AST and ALT levels than control animals (Fig. 4D, E). Likewise, proinflammatory cytokine levels in both liver tissue and serum were markedly reduced in the PTIR1-overexpressing group, indicating suppressed systemic inflammatory responses (Fig. 4F–I).

To investigate the molecular basis of PTIR1-mediated protection, we performed RNA sequencing (RNA-seq) on liver tissues from PTIR1-overexpressing and control mice. This transcriptomic analysis revealed broad gene-expression changes: 3639 genes were upregulated and 4535 were downregulated in PTIR1-overexpressing livers relative to controls (Supplementary Fig. 4A, B). Gene set enrichment analysis indicated that PTIR1 overexpression downregulated pathways involved in immune cell migration and activation (Fig. 4J, K), while gene sets associated with lipid metabolism were enriched in PTIR1-overexpressing livers (Supplementary Fig. 4C, D). Finally, RT-qPCR validation confirmed that PTIR1 dampens interferon-pathway signaling and attenuates inflammatory gene expression in the liver (Supplementary Fig. 4E–J). These findings support the conclusion that PTIR1 acts as a negative regulator of IFN-γ-driven hepatic inflammation in vivo.

### PTIR1 impairs host antiviral defense by suppressing type I interferon signaling

**Fig. 5:**
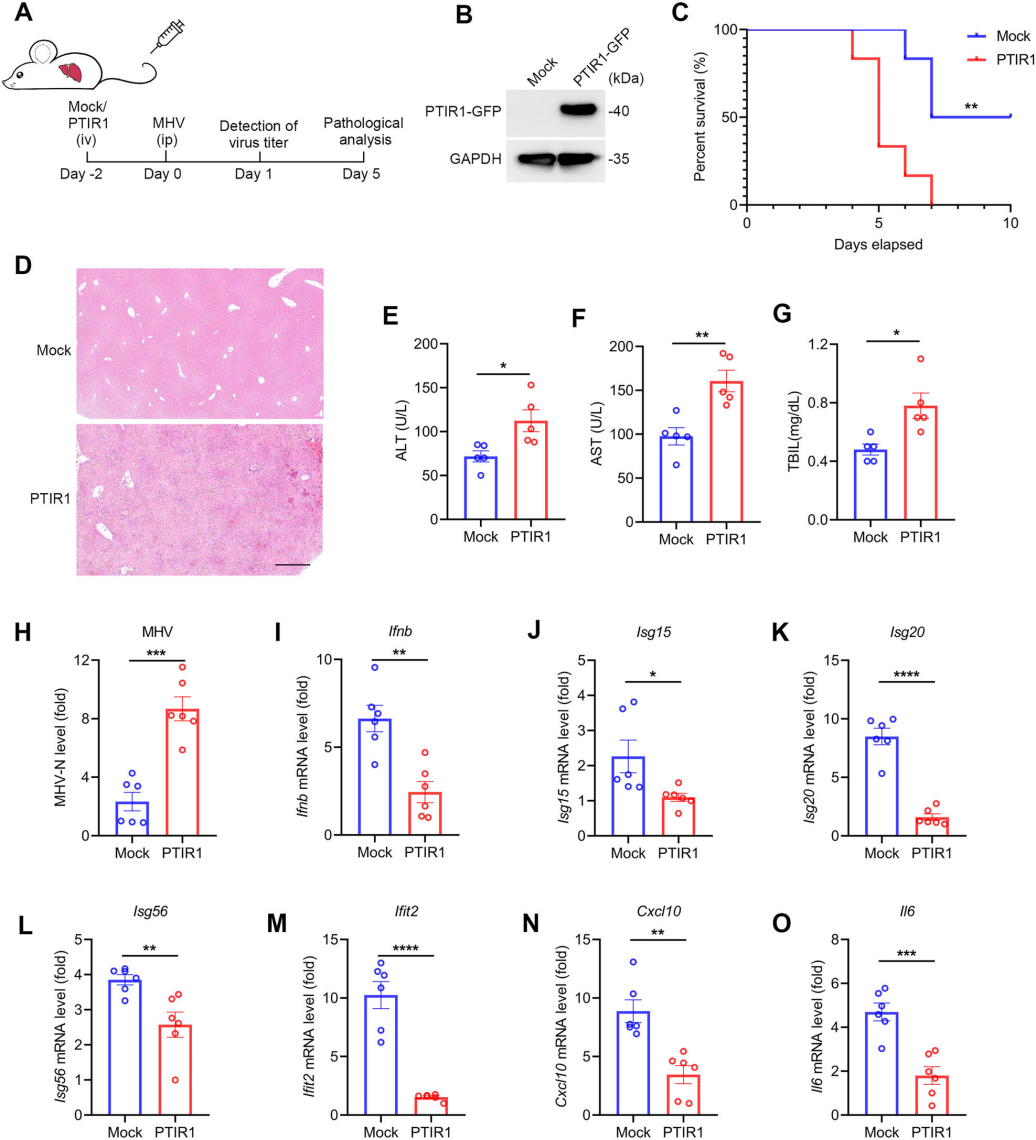
PTIR1 facilitates viral replication in vivo. **A** Graphic illustration of the study design for evaluating PTIR1 mediated antiviral effect in vivo. Mock or PTIR1 was first overexpressed in mouse liver through high-pressure tail-vein injection (iv) of indicated plasmids, and MHV-A59 was intraperitoneally injected (ip) to develop virus-infected mouse model. **B** Immunoblot analysis of protein expression level of PTIR1 in mice liver. **C** Survival analysis of mice with MHV infection (n = 5 mice, **P = 0.0084, log-rank (Mantel-Cox) test). **D** Histology analysis of mice livers under MHV-A59 infection for 5 days. The scale bars represent 500 μm. **E–G** After 5 days post infection, mice serum was collected and blood biochemistry test was performed. ALT, Alanine aminotransferase; AST, Aspartate aminotransferase; total bilirubin (TBIL) (n = 5, mean ± s.e.m., *P = 0.0195 (ALT), **P = 0.0039 (AST), **P = 0.0127 (TBIL), unpaired Student’s *t*-test). (**H**–**O**) RT-qPCR analysis of MHV-N as well as the mRNA level of ISGs in Mock or PTIR1-expressing mice livers at 24 hours post-MHV-A59 infection (n = 6 mice, mean ± s.e.m., *P = 0.0347, **P = 0.0015 (*Ifnβ*), **P = 0.0079 (*Isg56*), **P = 0.0014 (*Cxcl10*), ***P = 0.0001 (MHV-N), ***P = 0.0005 (*Il6*), ****P < 0.0001, unpaired Student’s *t*-test). The primers used for quantitative real-time PCR have been deposited in Supplemental Table 1.

To examine whether PTIR1-mediated suppression of IFN-I signaling impairs antiviral defense in vivo, we overexpressed PTIR1 or a control plasmid in mouse livers via high-pressure tail-vein injection. Mice were then challenged intraperitoneally with a lethal dose of murine hepatitis virus (MHV) (Fig. 5A, B). PTIR1-overexpressing mice exhibited significantly higher mortality following MHV challenge than control mice (Fig. 5C). Histological analysis of liver sections revealed more extensive hepatic tissue damage in PTIR1-overexpressing animals relative to controls (Fig. 5D). Consistent with this damage, serum AST, ALT, and total bilirubin levels were markedly elevated in these mice (Fig. 5E–G). Transcriptional profiling further showed that PTIR1 overexpression enhanced viral replication (Fig. 5H) while suppressing the induction of IFN-I and pro-inflammatory cytokines (Fig. 5I–O). Collectively, these data indicate that PTIR1 compromises antiviral immunity by dampening type I IFN responses.

### PTIR1 impairs RIG-I-mediated antiviral signaling

**Fig. 6:**
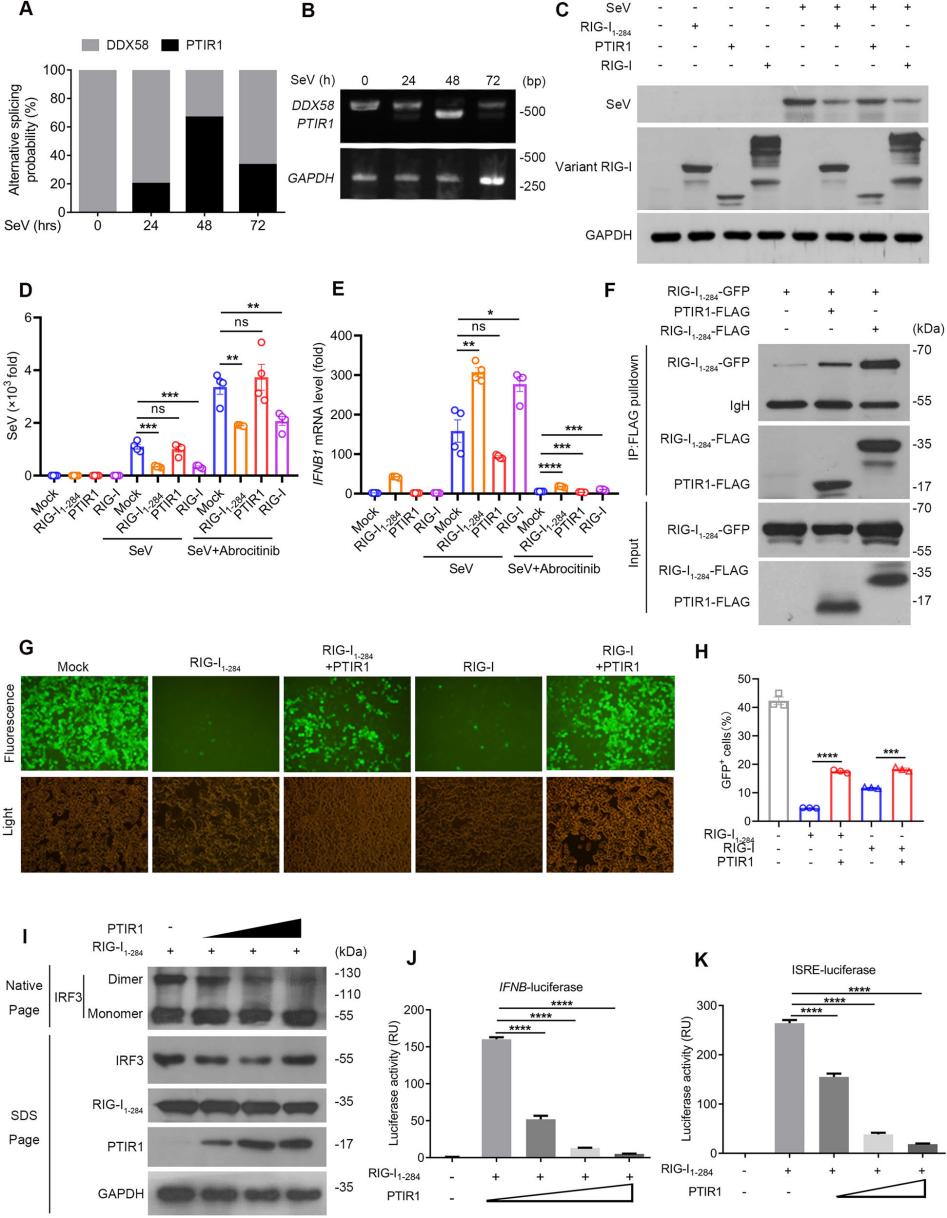
PTIR1 restricts RIG-I-mediated antiviral signaling. **A** Analysis of PTIR1 induction in HEK293T cells upon SeV infection. Total RNA of virus-infected HEK293T cells was extracted and converted into cDNA. *DDX58* transcript was amplified by PCR assay. PCR products were inserted in pGEM-T Easy vector and subjected to Sanger sequencing. Ratio of PTIR1 production was measured by clone number of pGEM-T Easy vector with PTIR1 insertion. **B** Expression of *DDX58* and *PTIR1* in HEK293T cells upon SeV infection. **C** Antiviral effects against SeV of RIG-I and PTIR1. HEK293T cells were treated with SeV at 24 h and viral titer was detected by anti-SeV antibody. **D**, **E** RT-qPCR analysis of Sendai virus (SeV) and *IFNB1* RNA levels in HEK293T cells with SeV infection with or without Abrocitinib treatment (n = 4 biological replicates, mean ± s.e.m., ns, not significant (*P* > 0.05), *P < 0.05, **P < 0.01, ***P < 0.001, ****P < 0.0001, unpaired Student’s *t*-test). **F** The interaction between PTIR1 and RIG-I_1-284_. HEK293T cells were transfected with indicated plasmids and subjected to immunoprecipitation with anti-FLAG antibody followed by western blot analysis. **G**, **H** HEK293T cells were transfected with indicated plasmids and then infected with VSV-GFP for 12 h. Images of GFP^+^ cells were shown in (**G**). Flow cytometric analysis of GFP^+^ cells was shown in (**H**) (n = 3 biological replicates, mean ± s.e.m., ***P = 0.0001, ****P < 0.0001, unpaired Student’s *t*-test). **I** Effect of PTIR1 on IRF3 dimerization. HEK293T cells were transfected with indicated plasmids and immunoblot analysis of IRF3 in dimer or monomer form was assessed by native PAGE. **J**, **K** Effect of PTIR1 on transactivation of *IFNB* and *ISRE*. HEK293T cells were transfected with vectors encoding active RIG-I truncation (RIG-I_1-284_) and/or PTIR1 plasmids together with IFNB or ISRE luciferase reporter plasmids. Data are presented in relative units (RU) relative to the activity of renilla luciferase (n = 3 biological replicates, mean ± s.e.m., ****P < 0.0001, unpaired Student’s *t*-test).

In addition to IFN stimulation, PTIR1 can also be induced by viruses at 24 h post-infection, peaks at 48 h, and then gradually declines (Fig. 6A, B). To examine whether PTIR1 participates in viral RNA sensing, we overexpressed vector control, PTIR1, full-length RIG-I, or RIG-I_1-284_ in HEK293T cells, followed by Sendai virus (SeV) infection. In contrast to full-length RIG-I and its active mutant, PTIR1 failed to elicit antiviral responses, indicating a loss of RNA-sensing capacity (Fig. 6C). To exclude the potential effect of STAT1 activation via the JAK-STAT pathway, we used Abrocitinib, a selective JAK1 inhibitor, and found that Abrocitinib treatment markedly reduced STAT1 phosphorylation, confirming effective inhibition of JAK-STAT signaling (Supplementary Fig. 5A). Under these conditions, PTIR1, in contrast to full-length RIG-I or the constitutively active RIG-I_1-284_ mutant, did not alter SeV titers at early infection stages regardless of Abrocitinib treatment (Fig. 6D). Notably, the inhibitory effect of PTIR1 on *IFNB1* expression became more pronounced in the presence of Abrocitinib (Fig. 6E), suggesting a possible role for PTIR1 in viral RNA recognition independent of downstream STAT1 signaling.

As RIG-I dimerization is essential for downstream signal transduction, we next assessed whether PTIR1 participates in this process. Co-IP assay showed that PTIR1 physically associates with RIG-I_1-284_ in transfected HEK293T cells, suggesting that it can engage in RIG-I dimerization (Fig. 6F). To evaluate the functional consequences of this interaction, we co-transfected a vector encoding PTIR1 or an empty vector with either full-length RIG-I or RIG-I_1-284_, followed by infection with VSV-GFP. PTIR1 overexpression significantly impaired RIG-I–mediated antiviral immunity (Fig. 6G, H). This suppression was further supported by reduced IRF3 dimerization (Fig. 6I), decreased IFNβ transcription (Fig. 6J and Supplementary Fig. 5B), and attenuated interferon-stimulated response element (ISRE) activation (Fig. 6K and Supplementary Fig. 5C) in both the presence and absence of Abrocitinib, confirming the inhibitory effect of PTIR1 on RIG-I-mediated innate immune signaling.

### PTIR1 restricts RIG-I ubiquitination and disrupts its association with MAVS

**Fig. 7:**
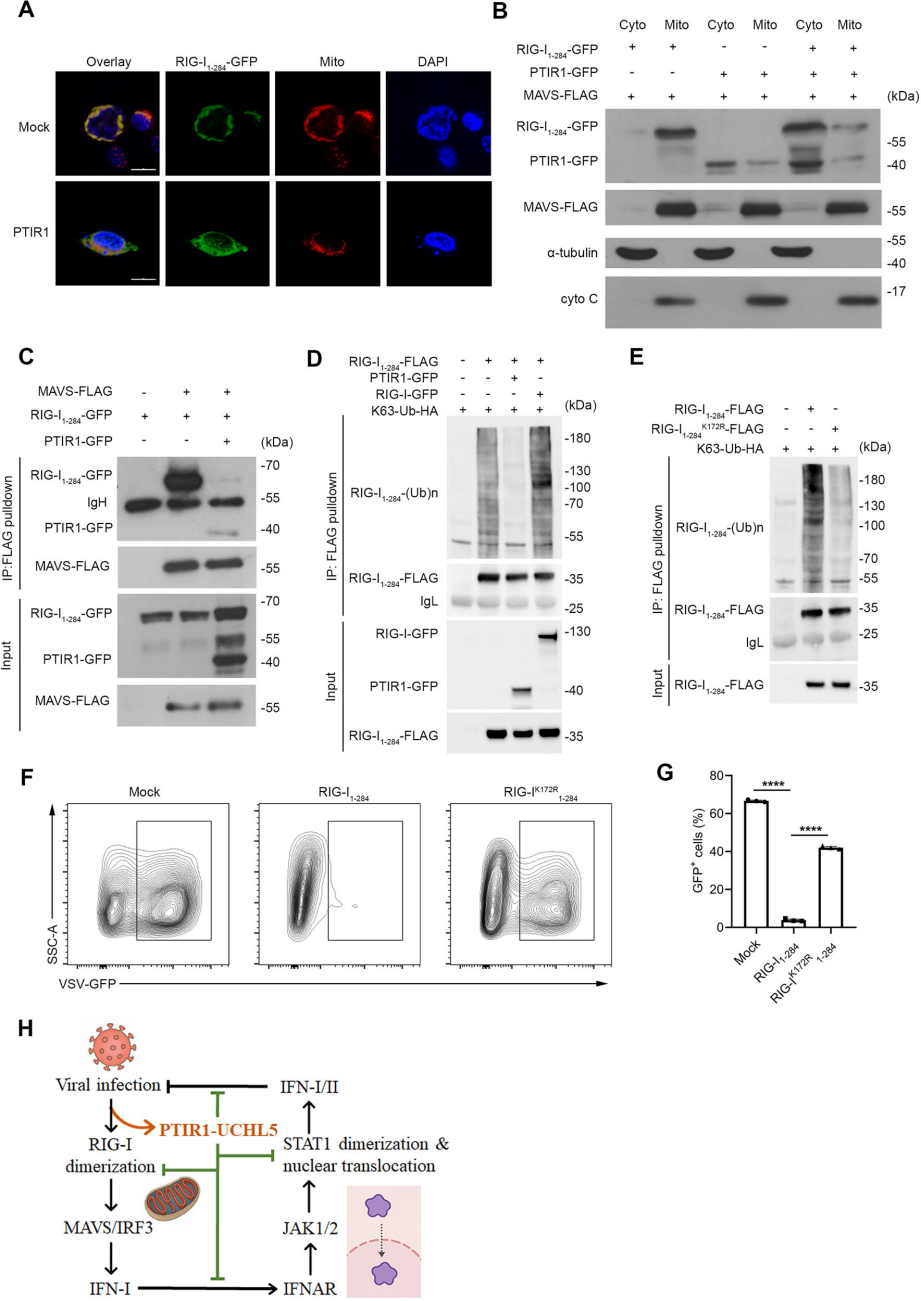
PTIR1 suppresses RIG-I ubiquitination and inhibits its association with MAVS. **A** Subcellular localization of GFP-tagged RIG-I_1-284_ in HEK293T cells with or without PTIR1 expression shown by confocal fluorescence microscopy. MitoTracker was used to indicate mitochondria. Overlay, merged images of GFP and MitoTracker. The scale bars represent 20 μm. **B** The role of PTIR1 in RIG-I_1-284_ subcellular localization. HEK293T cells were transfected with indicated plasmids, and subjected to mitochondrial extraction. Immunoblot analysis was performed to detect RIG-I_1-284_ location. **C** The interaction between RIG-I_1-284_ and MAVS with or without PTIR1. HEK293T cells were transfected with indicated plasmids and were subjected to immunoprecipitation with anti-FLAG antibody followed by western blot analysis. **D** Immunoprecipitation analysis of ubiquitination of RIG-I_1-284_ in HEK293T cells co-transfected with indicated plasmids. **E** Immunoprecipitation analysis of ubiquitination of WT or K172R mutant RIG-I_1-284_ in HEK293T cells transfected with indicated plasmids. **F**, **G** HEK293T cells were transfected with plasmids encoding WT or K172R mutant RIG-I_1-284_ and then infected with VSV-GFP for 12 h. Flow cytometric analysis of GFP^+^ cells was shown (n = 3 biological replicates, mean ± s.e.m., ****P < 0.0001, unpaired Student’s *t*-test). **H** Schematic diagram of the regulatory role of PTIR1 in host antiviral immunity and autoimmune disorders.

To investigate the molecular mechanism by which PTIR1 inhibits RIG-I signaling, we co-transfected the vector encoding RIG-I_1-284_ with either PTIR1 or a control vector in HEK293T cells. Confocal imaging revealed that PTIR1 overexpression markedly reduced mitochondrial localization of RIG-I_1-284_ (Fig. 7A). Subcellular fractionation confirmed this observation, showing that PTIR1 limits the accumulation of RIG-I_1-284_ in mitochondrial fractions and thereby reduces its likelihood of interacting with MAVS (Fig. 7B). Consistently, Co-IP assay demonstrated that PTIR1 significantly diminished the association between RIG-I_1-284_ and MAVS (Fig. 7C).

Given that the PTIR1-UCHL5 axis regulates STAT1 ubiquitination, we hypothesized that PTIR1 might also modulate RIG-I ubiquitination. Co-IP assay showed that PTIR1 overexpression markedly reduced ubiquitination of RIG-I_1-284_ (Fig. 7D). Mass spectrometry analysis further identified lysine 172 (K172) as a key site with reduced ubiquitination in the presence of PTIR1. To validate the importance of this site, we mutated K172 to arginine (K172R) and observed a substantial decrease in RIG-I_1-284_ ubiquitination (Fig. 7E). Notably, the K172R mutant also failed to mount an effective antiviral response, indicating a functional requirement for this modification (Fig. 7F, G). These results suggest that PTIR1 interferes with RIG-I signaling by restricting its ubiquitination at K172, thereby preventing MAVS engagement and downstream signal transduction.

## Discussion

In this study, we identify PTIR1, a primate-specific splice variant of *DDX58*, as a negative regulator of antiviral and inflammatory signaling. Unlike canonical RIG-I, PTIR1 lacks RNA-sensing capacity but exerts immunomodulatory functions by targeting two central nodes of innate immunity, STAT1 and RIG-I. We show that PTIR1 suppresses STAT1-dependent type I and II interferon signaling by promoting its deubiquitination at lysine 525 through the recruitment of UCHL5, thereby preventing STAT1 nuclear translocation. In parallel, PTIR1 restricts RIG-I ubiquitination at lysine 172 and disrupts MAVS engagement, effectively attenuating RIG-I-mediated antiviral responses. These dual mechanisms position PTIR1 as an inducible checkpoint that preserves tissue integrity during inflammation, while limiting the magnitude of antiviral immunity (Fig. 7H).

Type-I IFN plays a central role in establishing innate immunity, whereas type-II IFN predominantly mediates adaptive immunity against multiple pathogens [17]. Unlike IFN-I, which induces the formation of a STAT1-STAT2-IRF9 tri-complex, IFN-II stimulation primarily triggers STAT1 homo-dimerization [17]. Despite these differences, both complexes bind to unique regulatory DNA sequences, establishing functional redundancy in the antiviral response [18]. Our findings show that PTIR1 blocks STAT1 ubiquitination through activating the deubiquitinating enzyme UCHL5, which in turn impairs both homo- and hetero-dimerization of STAT1. Consequently, overexpression of PTIR1 ameliorates both IFN-I and IFN-II-mediated transcription of ISGs. These data suggest that PTIR1 not only modulates host innate antiviral immunity but may also play a role in the regulation of adaptive immune responses.

To explore the regulatory mechanisms by which type-I IFN signaling is orchestrated during viral infection, we infected HEK293T cells with Sendai virus (SeV). Upon pathogen recognition by PRRs, the mRNA level of *IFNβ* was upregulated, correlating with an increase in viral titers. This response was observed only during the early stage of viral infection (within 12 h post-infection). Despite a concomitant rise in viral titer and *DDX58* expression, IFNβ levels decreased gradually after 12 h. In contrast, *DDX58* expression remained elevated throughout this period, suggesting differential regulation between IFNβ and *DDX58* (Data not shown). The importance of IFN-I in the innate immune response is underscored by its dual role in protecting the host from pathogens and regulating immune homeostasis. However, overproduction of IFN-I is implicated in several autoimmune conditions and can promote chronic inflammation by enhancing the activation of immune cells. Thus, maintaining the balance of IFN-I activity is crucial for protecting against viral infection while preventing immune-related tissue damage. In this study, we identified PTIR1 can also be induced by pathogens at 24 h post-infection. We propose that PTIR1 functions as an immune regulator of IFN signaling and limits the risk of IFN-induced inflammatory damage.

In addition to phosphorylation, other unconventional PTMs, such as acetylation, methylation and SUMOylation, are involved in the regulation of STAT1 activity. Methylation of lysine 525 on STAT1 by methyltransferase SETD2 has been shown to be essential for IFN-I-induced gene transcription [11]. Interestingly, we identified poly-ubiquitination of STAT1 at lysine 525 during viral infection. PTIR1 blocks this ubiquitination by recruiting and activating UCHL5. Although both methylation and ubiquitination at K525 seem critical for STAT1 nuclear translocation, the deubiquitination on STAT1 K525 by PTIR1 preferentially impairs its homo- or hetero-dimerization with STAT2 rather than affecting STAT1 phosphorylation. Therefore, these two PTMs may regulate STAT1 activity through distinct mechanisms.

Our study also highlights a human-specific regulatory role of PTIR1 in the IFN response. Unlike murine cells, PTIR1 is selectively induced in human cells following IFN stimulation. The biological roles of interferons differ between species [19]. For instance, human IFNα enhances T cell polarization into Th1 and promotes IFNγ production, whereas murine IFNα fails to induce Th1 cells [20]. Furthermore, while murine IFNγ is protective in experimental autoimmune (allergic) encephalomyelitis, clinical trials have shown that supplementation of IFNγ exacerbates the disease in humans [21]. These differences suggest that human IFNs have stronger immunostimulatory effects, and excess IFN production can lead to immunopathology. We propose that PTIR1 functions as a human-specific suppressor of IFN signaling, mitigating the risk of IFN-induced inflammatory damage through deubiquitinating STAT1.

While our study provides mechanistic insight into PTIR1 function, several questions remain. First, the upstream signals controlling PTIR1 splicing and expression in specific immune cell types remain undefined. Second, whether PTIR1 is regulated dynamically in human inflammatory or infectious diseases is unknown. Finally, although PTIR1 is primate-specific, it remains to be determined whether analogous regulatory isoforms exist in other species or whether this represents a recent evolutionary adaptation to restrain excessive immunity.

In summary, our work uncovers PTIR1 as a previously unrecognized immune checkpoint that restrains STAT1- and RIG-I-mediated signaling through post-transcriptional and post-translational control. These findings not only provide a new framework for understanding how alternative splicing modulates innate immunity but also suggest potential therapeutic avenues for mitigating interferon-driven inflammation in autoimmunity and infection.

## Methods

### Cell lines

HEK293T cells were purchased from American Type Culture Collection (ATCC). Cells were cultured in Dulbecco’s Modified Eagle’s Medium (DMEM) supplemented with 10% Fetal Bovine Serum (FBS) and cells were negative for mycoplasma.

### Ethics approval

C57BL/6 mice were purchased from Vital River Laboratory Animal Technology. All animals were housed and maintained under specific pathogen-free conditions. All sex- and age-matched animal experiments were performed in accordance with protocols approved by the Ethics Committee of Peking University Health Science Center (approved number DLASBE0614).

### Virus infection

VSV (Vesicular Stomatitis Virus, Indiana strain) and SeV (Sendai Virus, Cantell strain) were gifts from Jun Zhang (Peking University). MHV-A59 was a gift from Fuping You (Peking University). For VSV and SeV infection, 0.1 multiplicity of infection (MOI) of virus was added to cell culture medium for indicated times. For in vivo viral infection, mice were intraperitoneally injected with 5 × 10^5^ pfu MHV-A59.

### S100-induced autoimmune hepatitis (AIH) model

The liver was perfused with PBS and fresh hepatic antigen S-100 was obtained by tissue homogenizing and centrifuging. Induction of AIH was performed via intraperitoneal injection of S100 protein (2.5 mg/250 μl) that was fully emulsified in an equal volume of complete Freund’s adjuvant every week.

### Concanavalin A (Con A)-induced AIH model

For induction of autoimmune hepatitis mice model, Con A (30 mg/kg body weight in normal saline) was administered intravenously in tail vein of the 6-week-old male mice with or without PTIR1 expression.

### Ischemia/reperfusion-induced acute kidney injury model

Mice were anesthetized and bilateral renal pedicles were clamped for 30 min. The clamps were then removed. 24 h after reperfusion, mice were killed, and mice serum and kidney were collected for follow-up experiments.

### PTIR1 detection

Cells were treated with indicated virus or cytokines and RNA was extracted using Trizol reagent (Invitrogen) and converted to cDNA with the Reverse Transcription System (Promega). The primers used to test PTIR1 were as follows: forward 5’-TTGGAGCTCCAGGAGGAA-3’, reverse 5’-CACAACCTGTAGGAGCACA-3’.

### Quantitative real-time PCR

Quantitative real-time PCR was performed as described [22] (all primers were listed in Supplemental Table 1).

### Luciferase reporter assay

Luciferase reporter assay was performed as described previously [23]. Briefly, HEK293T cells were transfected with various pGL3 luciferase reporter plasmids and lysed with passive lysis buffer (Promega). Luciferase activity was assessed by the Dual Luciferase Assay System (Promega).

### Co-immunoprecipitation and immunoblot analysis

Total cell lysates were prepared by co-immunoprecipitation lysis buffer (10% glycerol, 0.5% NP-40, 150 mM NaCl, 0.1 mM EDTA) with protease inhibitor (Roche) and then incubated with anti-FLAG antibody (F3165) and protein A/G (sc-2003) or S-tag beads at 4 °C. The precipitants were washed three times by PBS containing 0.1% NP-40 and then subjected to SDS-Page. Antibodies against DDX58 (sc-376845), Sendai (PD029C1), IRF3 (ab68481), ADAR1 (CST #14175S), STAT1 (CST #9172), phospho-STAT1 (Y701) (YP0249), FLAG (F3165), GFP (RM1008), HA (H3663), GAPDH (RM2002), α-tubulin (RM2007) and HDAC1 (sc-8410) were used.

### Mass spectrum

To analyze PTIR1 associated proteins, HEK293T cells transfected with PTIR1 were lysed with co-immunoprecipitation lysis buffer. Whole cell lysates with anti-FLAG M2 beads (F2426) were rotated for 4 h and 3×FLAG peptide (F4799) was used to elute the binding components. The samples were then subjected to NuPAGE 4–12% gels (Invitrogen) and Coomassie blue staining. After Coomassie blue staining of a gel, excised gel segments were subjected to in-gel trypsin digestion and dried. Peptides were dissolved in 10 μL 0.1% formic acid and auto-sampled directly onto a 100 μm × 10 cm fused silica emitter made in our laboratory packed with reversed-phase ReproSil-Pur C18-AQ resin (3 μm and 120 Å; Ammerbuch, Germany). Samples were then eluted for 50 min with linear gradients of 5–32% acetonitrile in 0.1% formic acid at a flow rate of 300 nl/min. Mass spectrometry data were acquired with an LTQ Orbitrap Elite mass spectrometer (Thermo Fisher Scientific) equipped with a nanoelectrospray ion source (Proxeon Biosystems). Fragmentation in the LTQ was performed by collision-induced dissociation (normalized collision energy, 35%; activation Q, 0.250; activation time, 10 ms) with a target value of 3000 ions. The raw files were searched with the SEQUEST engine against a database from the UniProt protein sequence database.

### Subcellular fractionation

Nuclear and cytoplasmic extracts were isolated with a nuclear-cytoplasmic extraction kit (Applygen, P1200) following the manufacturer’s protocol.

### Native PAGE

HEK293T cells were transfected with indicated plasmids and lysed with PBS containing 0.5% Triton X-100. Native sample buffer (250 mM Tris-HCl, 1% sodium deoxycholate, 0.5% bromophenol blue, 50% glycerol) was added to cell lysate, followed by native polyacrylamide gel [24].

### Immunofluorescence and confocal microscopy

HEK293T cells were transfected with the indicated plasmids and seeded on cover glass. After 12 h, cells were fixed with acetone and blocked by BSA. Primary antibodies were applied overnight and fluorophore-conjugated secondary antibodies were applied for 1 h, followed by fluorescence microscopy analysis.

### Statistical analysis

Statistical analysis was performed using the Prism GraphPad software v7.0. Differences between the two groups were analyzed using a two-tailed Student’s t test. *P* < 0.05 was considered significant.

## Supplementary information

Supplementary Data

Original Data

## Data Availability

The RNA-seq data has been deposited in the SRA database with the accession code PRJNA1222303.
