# Supplementary material for: Dual effects of the alternative spliced RIG-I isoform PTIR1 on host antiviral defense and immune homeostasis
Source: Cell Death Dis. 2025 Nov 10;16(1):821. doi: 10.1038/s41419-025-08159-x (PMC12603114; doi:10.1038/s41419-025-08159-x)
Supplement: Supplementary file 1 — Supplementary Data [file 41419_2025_8159_MOESM1_ESM.pdf]

Supplementary Materials for

Dual effects of the alternative spliced RIG-I isoform PTIR1 on host antiviral defense and immune homeostasis

Jia Song, Wenyu Tian, Lulu Liu, Xuyang Zhao, Wei Zhao*, Dan Lu*
Corresponding author: taotao@bjmu.edu.cn (Dan Lu), zhaowei@zryhyy.com.cn (Wei Zhao)

Supplemental Figure 1

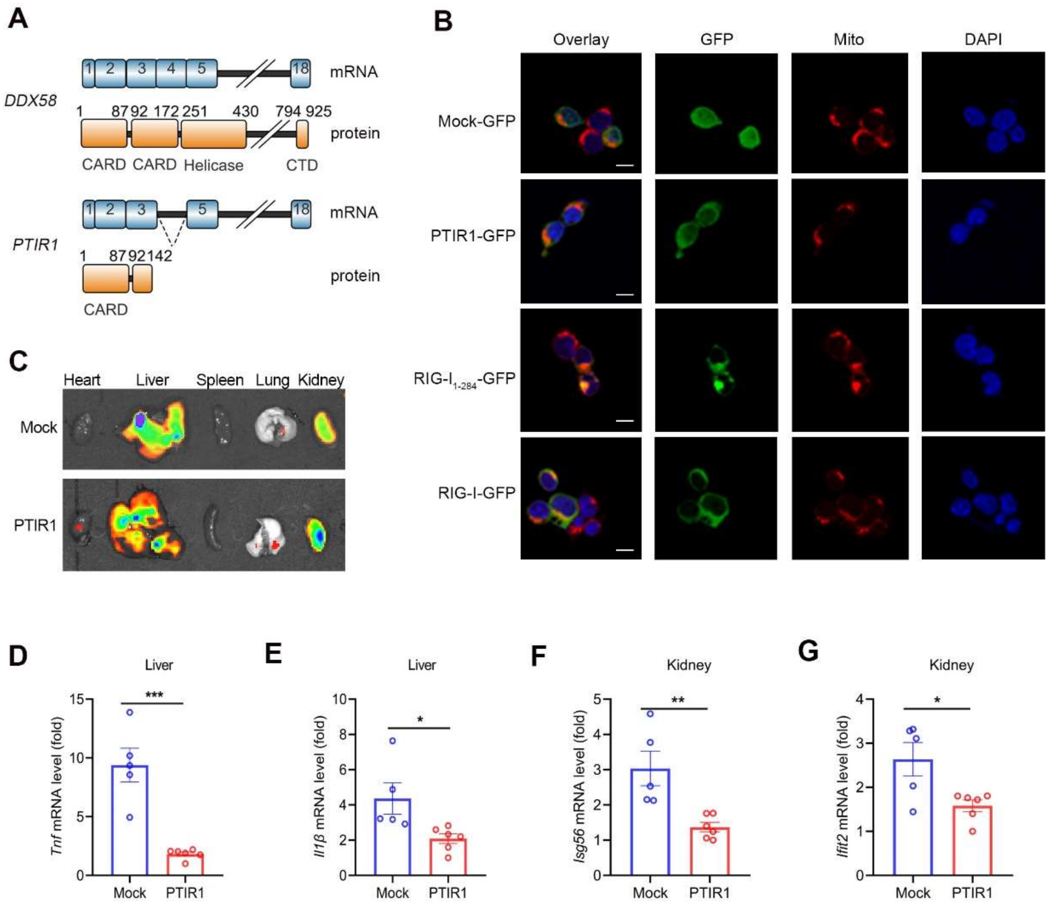

Supplemental Figure 1. PTIR1 inhibits host acute inflammation response

(**A**) Schematic presentation of mRNA and protein domain structure of DDX58 and PTIR1.

(**B**) Subcellular localization of GFP-tagged PTIR1, RIG-I$_{1-284}$ and RIG-I in HEK293T cells shown by confocal fluorescence microscopy. MitoTracker was used to indicate mitochondria. Overlay, merged images of GFP and MitoTracker. The scale bars represent 20 μm.

(**C**) *In vivo* expression of adenovirus-mediated protein expression measured by bioluminescence imaging.

(**D-E**) Quantitative real-time PCR analysis of the transcription of indicated genes in mice livers with or without PTIR1 24 hours after Con A administration (Mock, n = 5 mice; PTIR1, n = 6 mice,

mean ± s.e.m., **P* < 0.05, ****P* < 0.001, unpaired Student's t-test). The primers used for quantitative real-time PCR have been deposited in **Supplemental Table 1**.

(**F-G**) Quantitative real-time PCR analysis of the transcription of indicated genes in mice kidneys with or without PTIR1 24 hours after AKI (Mock, n = 5 mice; PTIR1, n = 6 mice, mean ± s.e.m., **P* < 0.05, ***P* < 0.01, unpaired Student's t-test). The primers used for quantitative real-time PCR have been deposited in **Supplemental Table 1**.

Supplemental Figure 2

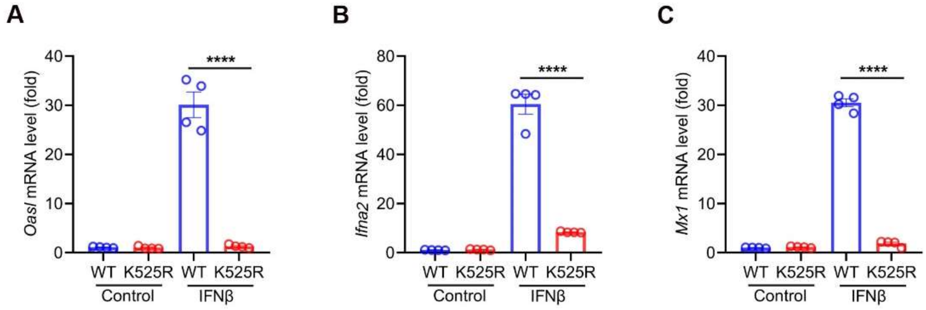

Supplemental Figure 2. Suppressive effect of PTIR1 on IFN signaling.

(**A-C**) Quantitative real-time PCR analysis of indicated genes in BMDM cells expressing STAT1^{WT} or STAT1^{K525R} with or without IFNβ (100 ng/mL) stimulation for 24 hours (n = 4 biological replicates, mean ± s.e.m., ****P < 0.0001, unpaired Student's t-test). The primers used for quantitative real-time PCR have been deposited in **Supplemental Table 1**.

Supplemental Figure 3

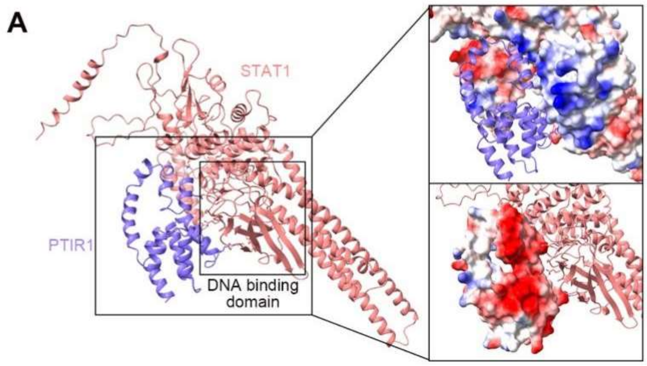

Supplemental Figure 3. Structure analysis of association of PTIR1 with STAT1.

(**A**) AlphaFold3 was used to analyze STAT1/PTIR1 complex.

Supplemental Figure 4

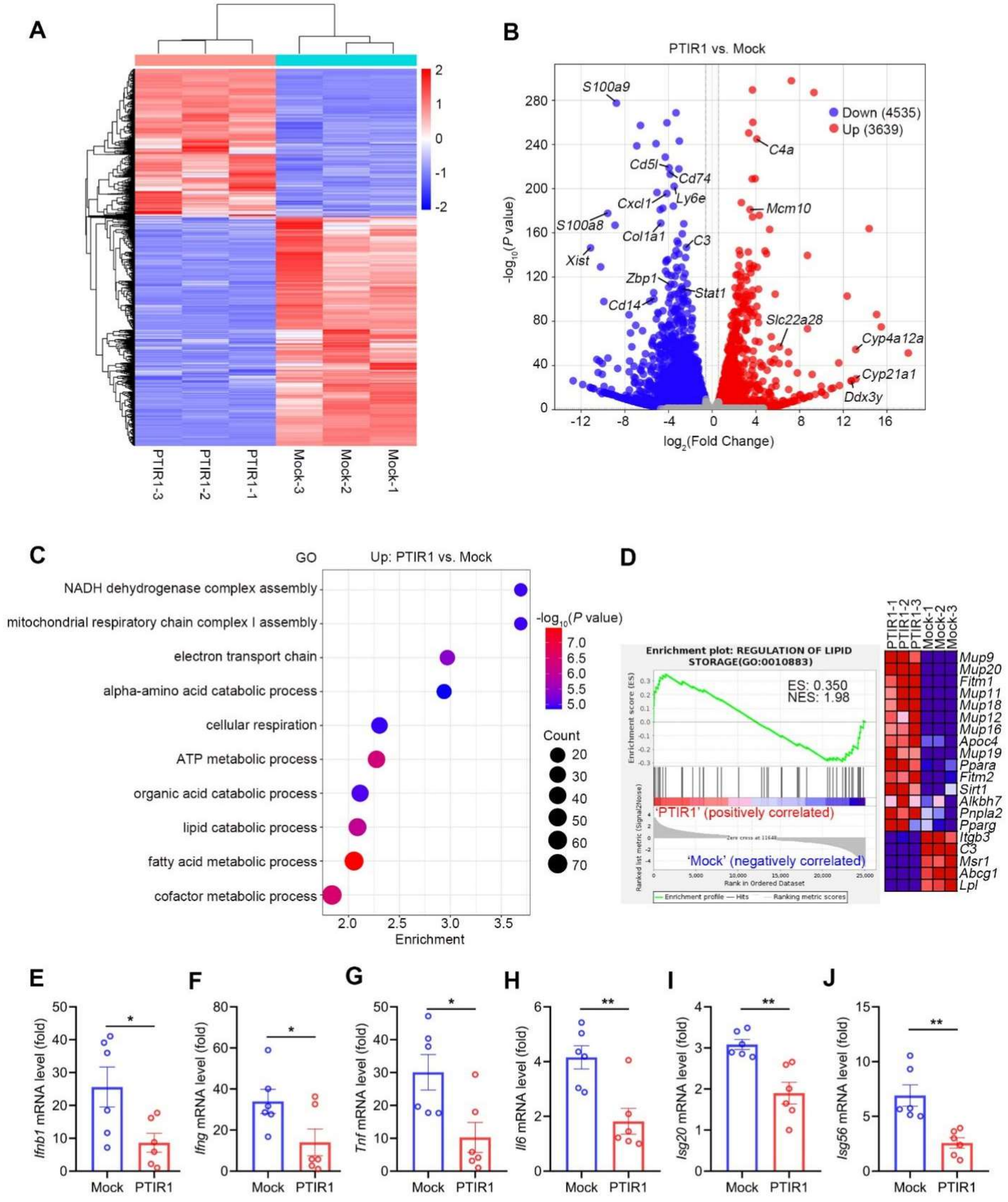

Supplemental Figure 4. PTIR1 inhibits inflammatory response in mouse liver.

(**A**) Heatmap of gene expression of mice livers with or without PTIR1 expression is presented (n = 3 biological replicates).

(**B**) Volcano plot analysis of pairwise comparison of transcriptional analysis between Mock and PTIR1 group.

(**C**) Genes that were significantly upregulated in PTIR1 expressing liver, compared with Mock liver, were analyzed using DAVID with GO terms.

(**D**) GSEA of genes expressed in mouse liver in AIH model, in the presence or absence of PTIR1 (n = 3 biological replicates). ES, enrichment score; NES, normalized enrichment score.

(**E-J**) RT-qPCR analysis of the mRNA level of inflammatory cytokines Mock or PTIR1-expressing liver (n = 6 biological replicates, mean ± s.e.m., **P* = 0.0305 (*Ifnb1*), **P* = 0.0463 (*Ifng*), **P* = 0.0184 (*Tnf*), ***P* = 0.0042 (*Il6*), ***P* = 0.0023 (*Isg20*) and ***P* = 0.0026 (*Isg56*), unpaired Student's t-test).

Supplemental Figure 5

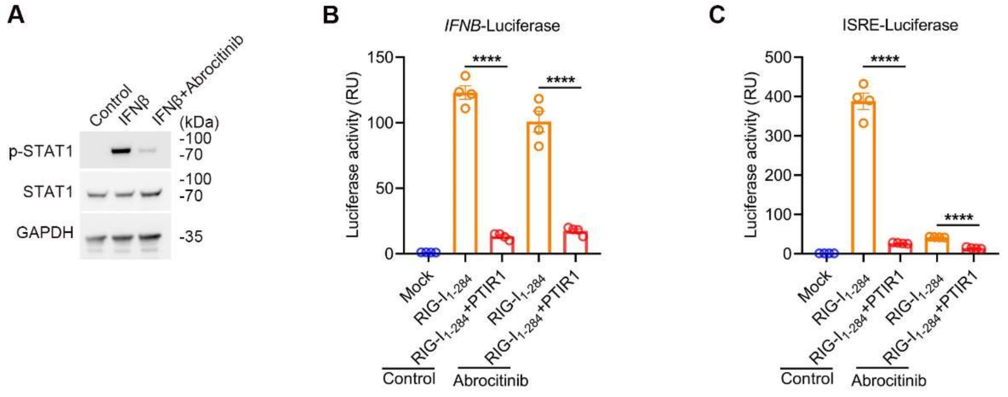

Supplemental Figure 5. PTIR1 restricts RIG-I-mediated antiviral signaling.

(**A**) Phosphorylation of STAT1 in HEK293T cells upon IFNβand Abrocitinib treatment, tested by western blot with anti-phospho-STAT1 antibody.

(**B-C**) Effect of PTIR1 on transactivation of *IFNB* and *ISRE* with or without Abrocitinib treatment. HEK293T cells were transfected with vectors encoding active RIG-I truncation (RIG-I$_{1\text{-}284}$) or/and PTIR1 plasmids together with IFNB or ISRE luciferase reporter plasmids. Data are presented in relative units (RU) relative to the activity of renilla luciferase (n = 4 biological replicates, mean ± s.e.m., *****P* < 0.0001, unpaired Student's t-test).

Supplemental Table 1. The primers used for quantitative real-time PCR.

Primer name	Sequence (5'->3') -Forward	Sequence (5'->3') -Reverse
SeV	CAAAAGTGAGGGCGAAGGAGAA	CGCCCAGATCCTGAGATACAGA
MHV-N	CAGATCCTTGATGATGGCGTAGT	AGAGTGTCCTATCCCGACTTTCTC
Ifnb1	CAGCTCCAAGAAAGGACGAAC	GGCAGTGTAACTCTTCTGCAT
Ifng	ATGAACGCTACACACTGCATC	CCATCCTTTTGCCAGTTCCTC
Il1β	GCAACTGTTCCTGAACTCAACT	ATCTTTTGGGGTCCGTCAACT
Tnf	CCCTCACACTCAGATCATCTTCT	GCTACGACGTGGGCTACAG
Cxcl10	CCAAGTGCTGCCGTCATTTTC	GGCTCGCAGGGATGATTTCAA
Ifitm2	TGGGCTTCGTTGCCTATGC	AGAATGGGGTGTTCTTTGTGC
Ifi44	AACTGACTGCTCGCAATAATGT	GTAACACAGCAATGCCTCTTGT
Ifih1	AGATCAACACCTGTGGTAACACC	CTCTAGGGCCTCCACGAACA
Oasl	CAGGAGCTGTACGGCTTCC	CCTACCTTGAGTACCTTGAGCAC
Mx1	GACCATAGGGGTCTTGACCAA	AGACTTGCTCTTTCTGAAAAGCC
Ifna2	TACTCAGCAGACCTTGAACCT	CAGTCTTGGCAGCAAGTTGAC
Il6	TAGTCCTTCCTACCCCAATTTCC	TTGGTCCTTAGCCACTCCTTC
Isg20	TGGGCCTCAAAGGGTGAGT	CGGGTCGGATGTACTTGTCATA
Ifit2	AGTACAACGAGTAAGGAGTCACT	AGGCCAGTATGTTGCACATGG
Isg15	GGTGTCCGTGACTAACTCCAT	TGGAAAGGGTAAGACCGTCCT
Isg56	CTGAGATGTCACTTCACATGGAA	GTGCATCCCCAATGGGTTCT
Actb	GAGACCTTCAACACCCCAGC	ATGTCACGCACGATTTCCC
IFIT2	GGGCAGAGAACAGGAAGATAC	TATGAGGAGGGCAGAGTAGAG

ISG56	CCAACAGTGTAGTAGCCTCAAA	GGTGCGTCCTTAGAAGAAAGAG
HLA-A	GAGGAGGAAGAGCTCAGATAGA	GGCAGCTGTCTCACACTTTA
HLA-C	AATGTGAGGAGGTGGAGAGA	CCTCTCTGGAACAGGAAAGATG
IFNB1	GCTTGGATTCCTACAAAGAAGCA	ATAGATGGTCAATGCGGCGTC
GAPDH	ACCCACTCCTCCACCTTTGA	CTGTTGCTGTAGCCAAATTCGT
